# Does First Blood Draw Matter in Retinopathy of Prematurity: A Comparative Analysis of Inflammation and Hypoxia-Related Parameters

**DOI:** 10.22336/rjo.2025.61

**Published:** 2025

**Authors:** Ceren Durmaz Engin, Ozlem Ozkan, Taylan Ozturk

**Affiliations:** 1 Department of Ophthalmology, Izmir Democracy University Buca Seyfi Demirsoy Education and Research Hospital, Izmir, Turkey; 2 Department of Biomedical Technologies, Dokuz Eylul University, Izmir, Turkey; 3 Department of Ophthalmology, Dokuz Eylul University School of Medicine, Izmir, Turkey; 4 Department of Ophthalmology, Tinaztepe University School of Medicine, Izmir, Turkey

**Keywords:** complete blood count, hemoglobin albumin lymphocyte and platelet score, pan-immune inflammation value, retinopathy of prematurity, systemic immuno-inflammatory index, systemic inflammatory response index, ABG = Arterial Blood Gas, APROP = Aggressive Posterior Retinopathy of Prematurity, AUC = Area Under the Curve, BW = Birth Weight, CBC = Complete Blood Count, DED = Dry Eye Disease, DRP = Diabetic Retinopathy, GA = Gestational Age, HALP = Hemoglobin, Albumin, Lymphocyte, and Platelet (score), Hb / HGB = Hemoglobin, ICROP = International Classification of Retinopathy of Prematurity, IVH = Intraventricular Hemorrhage, LP = Laser Photocoagulation, LMR = Lymphocyte-to-Monocyte Ratio, MCV = Mean Corpuscular Volume, MCHC = Mean Corpuscular Hemoglobin Concentration, NEC = Necrotizing Enterocolitis, NICU = Neonatal Intensive Care Unit, NLR = Neutrophil-to-Lymphocyte Ratio, NO = Nitric Oxide, OR = Odds Ratio, PIV = Pan-Immune-Inflammation Value, PLR = Platelet-to-Lymphocyte Ratio, pO_2_ = Partial Pressure of Oxygen, pCO_2_ = Partial Pressure of Carbon Dioxide, PVL = Periventricular Leukomalacia, ROC = Receiver Operating Characteristic, ROP = Retinopathy of Prematurity, RBC = Red Blood Cell, SII = Systemic Immune-Inflammation Index, SIRI = Systemic Inflammatory Response Index

## Abstract

**Aim:**

To compare the inflammation and hypoxia-related parameters in the first 24-hour complete blood count (CBC) and arterial blood gas (ABG) analysis among infants diagnosed with retinopathy of prematurity (ROP), those requiring treatment for ROP, and those without any stage of ROP.

**Methods:**

Three hundred and thirty infants screened for ROP with CBC, ABG analysis, and albumin results in the first 24 hours after delivery were included. In addition to individual cell counts on CBC and hypoxia indicators in ABG analysis, systemic immuno-inflammatory index (SII), systemic inflammatory response index (SIRI), pan-immune inflammation value (PIV), and hemoglobin, albumin, lymphocyte, and platelet (HALP) values were calculated and compared between the study groups.

**Results:**

No significant differences were observed in SII, SIRI, PIV, and HALP scores among any groups, whether for ROP diagnosis or treatment requirement. Multivariate analysis showed significant differences in red blood cell count (RBC) (p=0.001), hemoglobin (HGB) (p=0.001), mean corpuscular volume (p<0.001), and mean corpuscular hemoglobin concentration (p<0.001) between infants with and without ROP. RBC (p=0.001) and HGB (p=0.002) also significantly differed between neonates needing treatment and those not, but were not independent predictors of treatment necessity in the multivariate analysis. No significant differences in ABG parameters were found among the groups for either ROP diagnosis or treatment need.

**Discussion:**

This study contributes to the literature by being the first to evaluate emerging inflammatory indices such as SIRI, PIV, and HALP in the context of ROP, demonstrating that these parameters from the initial blood draw do not provide predictive value. While erythrocyte-related markers showed some associations, they were not independent predictors of treatment need, highlighting the importance of cautious interpretation of early hematologic findings. These results underscore that the very first blood sample may reflect transitional neonatal physiology more than disease-specific mechanisms, and they encourage future studies to explore longitudinal sampling for more reliable biomarkers.

**Conclusion:**

The novel inflammatory markers, SII, SIRI, PIV, and HALP score, obtained from blood tests within the first 24 hours of neonatal life, do not appear to be predictive of ROP development or the need for treatment.

## Introduction

Retinopathy of Prematurity (ROP) is a vision-threatening condition predominantly affecting premature infants, characterized by the abnormal proliferation of retinal blood vessels, which can lead to retinal detachment and potential blindness if untreated [1]. The incidence of ROP has risen notably in recent years, mainly due to the better survival rates of premature newborns, highlighting its significance as a public health issue. The development of ROP involves multiple factors, including oxygen levels, vascular growth factors, and inflammatory cytokines, as well as cells, which collectively contribute to retinal ischemia, subsequent neovascularization, and ultimately proliferative retinopathy [2]. Multiple studies have demonstrated increased levels of pro-inflammatory cytokines in the vitreous humor and serum of infants diagnosed with ROP, highlighting the significant role of both localized and systemic inflammation in its pathogenesis [3-6]. Another crucial factor in ROP development is exposure to hypoxia followed by hyperoxia, which places preterm infants at heightened risk for oxidative stress and associated injuries due to underdeveloped antioxidative defense mechanisms [7]. Saugstad introduced the term “Oxidative Radical Disease of Neonatology” to describe various morbidities linked to free radicals in preterm infants, such as necrotizing enterocolitis (NEC), intraventricular hemorrhage (IVH), periventricular leukomalacia (PVL), and ROP [8]. Not only the long-term irregularities of blood gases but also the abnormally high and low levels of carbon dioxide and oxygen during the first few hours after birth have been linked to increased inpatient mortality and the occurrence of comorbidities, including IVH and PVL, in very preterm infants [9-11].

Screening for ROP typically relies on gestational age (GA) and birth weight (BW) [12]. However, extensive research has identified a range of additional risk factors that may contribute, including genetic, maternal, prenatal, perinatal, and nutritional factors, as well as issues associated with medical treatments and other prematurity-related comorbidities. Early blood count parameters also emerge as significant, given their roles in blood gas exchange, immunomodulation, and antioxidant defense. Studies assessing individual cell counts at various points in the neonatal period have produced inconsistent findings [13,14]. Furthermore, specific inflammatory markers derived from the complete blood count (CBC), such as the neutrophil-to-lymphocyte ratio (NLR), lymphocyte-to-monocyte ratio (LMR), and platelet-to-lymphocyte ratio (PLR), have been investigated in ROP, showing variable results [15-17].

Recently, emerging inflammatory markers such as the Systemic Immune Inflammation Index (SII), Systemic Inflammatory Response Index (SIRI), Pan-immune Inflammation Value (PIV), and Hemoglobin, Albumin, Lymphocyte, and Platelet (HALP) score, which encompass various subsets of white blood cells and proteins, have been shown to reflect the interplay between inflammation and immune response. These markers can be derived through straightforward computational formulas. Recent studies have established a link between these indicators and diseases such as cancer, cardiovascular disease, and diabetic nephropathy [18-20]. A limited number of studies have also evaluated these biomarkers in ophthalmic diseases, including dry eye disease (DED), diabetic retinopathy (DRP), and endophthalmitis, all of which are associated with systemic inflammation [21-23]. To our knowledge, only two studies have investigated the role of SII specifically in ROP patients; one reported a significant difference in SII levels within the first 24 hours between ROP and non-ROP patients, while the other did not find such a difference [24,25]. As of the current literature, the roles of SIRI, PIV, and HALP in ROP remain unexplored. Therefore, this study aims to evaluate the potential of these emerging inflammatory markers, along with CBC and arterial blood gas (ABG) related risk factors collected within the first 24 hours of neonatal life, in predicting the development of ROP and the necessity for treatment.

## Materials and methods

### Study Population

A retrospective review of the medical records of 2440 preterm infants who were screened for ROP at our tertiary referral center over the past decade, from September 2010 to September 2020, was conducted. The minimum sample size for both ROP and non-ROP groups was established after performing a power analysis using G*Power 3.1.9.7 software (Faul, Erdfelder, Lang & Buchner). Infants with conditions such as blood culture-confirmed sepsis, necrotizing enterocolitis, any hematological disorders, congenital anomalies, or severe illnesses affecting inflammatory responses were excluded. Additionally, infants who had received blood transfusions, postnatal steroids, or were born within 48 hours following the administration of antenatal steroids to the mother were also excluded. Further exclusions were made for neonates lacking complete medical records for study parameters, including the results of CBC, albumin, and ABG tests within the first 24 hours post-delivery. Ultimately, the study included data from 330 infants who were admitted to the neonatal intensive care unit (NICU) and had complete early blood test results. This study adhered to the Declaration of Helsinki guidelines, received approval from the institutional Ethics Committee, and all participating parents provided written informed consent for their infants’ involvement.

### ROP screening and treatment

ROP screenings were carried out by an experienced ophthalmologist (T.O.) using the International Classification of ROP (ICROP) guidelines to document the disease stage, zone location, and any indications of plus disease. Preterm infants born at a GA ≤ 32 weeks or BW ≤ 1500 g were screened for ROP. The screening program also included preterm infants who needed cardiopulmonary support or were identified by their neonatologist as being at risk for ROP, regardless of their BW or GA. Screenings were initially set to begin at 4 weeks after birth, provided the postmenstrual age was at least 31 weeks. Treatment with LP was administered for cases exhibiting aggressive posterior disease (APROP) or those classified as Type 1 ROP, which included zone I, any stage with plus disease; zone I, stage 3 without plus disease; or zone II, stage 2 or 3 with plus disease. Infants diagnosed with ROP were subsequently re-examined periodically, ranging from a few days to three weeks, until the ophthalmologist decided they could be discharged from screening. Post-discharge, all patients were scheduled for routine eye exams at the age of one year. The recorded data reflected the most advanced stage of ROP observed in the eye.

### Blood analysis results and inflammatory indices

The initial 24-hour results for CBC, albumin, and ABG were retrospectively examined and documented. If these tests were conducted multiple times within the first day, only the initial results were considered. The analysis included individual cell counts, as well as NLR and PLR, which were calculated by dividing the absolute counts of neutrophils and platelets by the absolute lymphocyte count, respectively. The ABG analysis assessed pH, pCO2, pO2, HCO3, base deficit, and lactate levels. Additionally, several novel inflammatory markers were calculated as follows:

- Systemic immune-inflammation index (SII): [platelets (/L) x neutrophils (/L)] / lymphocytes (/L)
- Systemic inflammation response index (SIRI): [neutrophils (/L) × monocytes (/L)] / lymphocytes (/L)
- Pan-Immune-Inflammation Value (PIV): [neutrophils (/L) × platelets (/L) x monocytes (/L)] / lymphocytes (/L)
- HALP score: [hemoglobin (g/L) × albumin (g/L) × lymphocytes (/L)] / platelets (/L)

### Statistical analysis

Statistical analyses were executed using SPSS software version 25.0 (IBM, Armonk, NY). The normality of data distributions was assessed with the Kolmogorov-Smirnov test. For descriptive statistics, we used measures including mean, standard deviation, median, minimum, and maximum. Differences in variables between ROP and no-ROP groups, as well as between treatment-positive and treatment-negative groups, were analyzed using the independent sample t-test. Categorical variables were quantified using frequency and percentage, and comparisons between these groups were made using the Chi-square test. The variables significantly associated with the development or treatment need of ROP were included in a multivariate logistic regression model, from which odds ratios (ORs) and 95% confidence intervals (CIs) were derived. The diagnostic value of significant parameters in multivariate analysis was determined through Receiver Operating Characteristic (ROC) curve analysis. Results with a p-value less than 0.05 were deemed statistically significant.

## Results

A total of 2440 preterm infants were screened for ROP during the study period. Of these, 330 (166 males and 164 females) met the inclusion criteria and were enrolled. The mean GA and BW of the enrolled infants were 30.15 ± 3.8 weeks (range, 23–40 weeks) and 1457.2 ± 657.8 g (range, 463–3670 g), respectively. Details on the number of infants diagnosed with any stage of ROP and those who received treatment are provided in **Table 1**, along with other demographic and clinical characteristics.

**Table 1:** Demographics and clinical characteristics of the study group

Variable	ROP (+) Prematures (n=104)	ROP (-) Prematures (n=226)	p value
GA, weeks	27.41 ± 3.07	31.41 ± 3.43	**<0.001** *
BW, g	993.98 ± 447.44	1670.39 ± 629.48	**<0.001** *
Maternal age	29.74 ± 5.52	30.58 ± 6.65	0.265 *
APGAR1	4.53 ± 2.29	6.34 ± 2.11	**<0.001** *
APGAR5	6.36 ± 1.98	7.93 ± 1.79	**<0.001** *
Gender, males (%)	44 (42.3%)	120 (53.0%)	0.068 **
Multiple pregnancy, n (%)	30 (28.8%)	68 (30.0%)	0.821 **
Gestational DM, (%)	15 (14.4%)	48 (21.2%)	0.177 **
Gestational HT, (%)	11 (10.5%)	27 (11.9%)	0.143 **

BW = birth weight; DM = diabetes mellitus; GA = gestational age; HT = hypertension

* Independent samples t-test

** Chi-square test

**Fig. 1:**
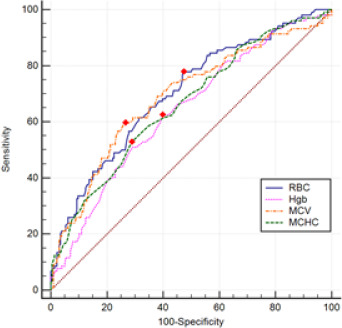
ROC curve analysis of significant CBC parameters from multivariate analysis for ROP development. Red dots indicate the cut-off values for each parameter

Significant differences in red blood cell (RBC), hemoglobin (HGB), mean corpuscular volume (MCV), and mean corpuscular hemoglobin concentration (MCHC) were observed between neonates diagnosed with ROP and those without. Conversely, no significant differences were found in NLR, PLR, SII, SIRI, PIV, and HALP scores between the two groups (p>0.05 for all). CBC parameters, albumin, and inflammatory indices for neonates diagnosed with ROP and those without are detailed in **Table 2**. Multivariate logistic regression analysis was used to adjust for confounding factors and revealed that RBC (p=0.001), HGB (p=0.001), MCV (p<0.001), and MCHC (p<0.001) remain significant in differentiating between ROP-positive and ROP-negative infants. At the same time, platelet and albumin did not retain significance, although they were significantly different between the two groups in univariate analysis. GA was also identified as an independent risk factor for ROP. The results of the multivariate logistic regression analysis for ROP development are presented in **Table 3. Fig. 1** shows the ROC curves for significant variables in multivariate analysis for ROP development. Postnatal first-day RBC of 4.35 or higher predicted ROP development with 78% sensitivity and 53% specificity; the area under the curve was 0.700 (p<0.001; 95% CI: 0.640–0.761). Postnatal first-day HbG value of 15.65 or higher was considered as a predictor for ROP development with 62% sensitivity and 61% specificity, and the area under the curve was 0.640 (p<0.001; 95% CI: 0.576–0.703). The area under the curve for MCV in the postnatal first month was 0.689, and the MCV value of 115.75 or higher predicted the development of ROP with a sensitivity of 59% and a specificity of 74% (p<0.001; 95% CI: 0.625–0.752). Postnatal first-day MHCH value of 33.05 or more was considered a risk factor for ROP development with 53% sensitivity and 71% specificity (p<0.001; 95% CI:0.598–0.725), and the area under the curve was 0.662.

**Table 2:** Comparison of blood parameters between neonates with and without ROP (Mean ± SD)

	ROP (+) Prematures (n=104)	ROP (-) Prematures (n=226)	p value*
RBC (10^3^ μL)	3.89 ± 0.77	4.46 ± 0.73	**<0.001**
HGB (g/dL)	14.88 ± 2.80	16.25 ± 2.71	**<0.001**
HCT (%)	48.55 ± 32.11	49.69 ± 9.06	0.620
MCV (fL)	117.60 ± 10.38	112.04 ± 8.08	**<0.001**
MCHC (g/dL)	33.19 ± 2.06	32.10 ± 1.51	**<0.001**
RDW (%)	18.29 ± 2.13	18.33 ± 2.19	0.883
WBC (10^3^ μL)	13.48 ± 12.28	12.42 ± 11.82	0.461
NEU (10^3^ μL)	7.71 ± 9.39	6.52 ± 6.14	0.238
LYM (10^3^ μL)	4.07 ± 4.36	3.98 ± 2.48	0.811
MONO (10^3^ μL)	1.13 ± 0.93	1.01 ± 0.81	0.236
PLT (10^3^ μL)	181.94 ± 92.33	213.53 ± 108.85	**0.010**
PCT (%)	0.16 ± 0.17	0.16 ± 0.08	0.853
ALBUMIN (g/dL)	2.89 ± 0.49	3.16 ± 0.48	**<0.001**
NLR	2.22 ± 2.18	2.04 ± 2.52	0.535
PLR	61.81 ± 46.91	70.88 ± 56.69	0.155
SII	443.90 ± 525.52	442.77 ± 557.71	0.986
SIRI	3.34 ± 5.53	2.41 ± 3.71	0.119
PIV	716.94 ± 1396.64	563.05 ± 1128.39	0.325
HALP score	14.40 ± 23.03	14.28 ± 18.31	0.956

HALP score = hemoglobin, albumin, lymphocyte and platelet score; HGB = hemoglobin; HCT = hematocrit; LYM = lymphocyte; MCHC = mean corpuscular hemoglobin concentration; MCV = mean corpuscular volume; MONO = monocyte; NEU = neutrophil; NLR = neutrophil-to-lymphocyte ratio; PIV = pan-immune-inflammation value; platelet indices variation; PLR = platelet-to-lymphocyte ratio; PLT = platelet; PCT = plateletcrit; RBC = red blood cell; RDW = red cell distribution width; SII = systemic immune-inflammation index; SIRI = systemic inflammation response index; WBC = white blood cell

* Independent samples t-test; significant p-values are given in bold.

**Table 3:** Multivariate analysis of the predictors for ROP development

Variable	OR	95% CI		p value
		Lower	Upper	
GA, weeks	0.837	0.703	0.996	**0.045**
BW, g	0.999	0.998	1.000	0.075
APGAR1	0.914	0.706	1.183	0.496
APGAR5	1.089	0.808	1.468	0.577
RBC (10^3^ μL)	141.853	6.670	3016.643	**0.001**
HGB (g/dL)	0.240	0.106	0.545	**0.001**
MCV (fL)	1.215	1.093	1.350	**0.000**
MCHC (g/dL)	2.340	1.575	3.474	**0.000**
PLT (10^3^ μL)	0.997	0.993	1.001	0.125
ALBUMIN (g/dL)	0.932	0.456	1.907	0.848

BW = birth weight; GA = gestational age; HGB = hemoglobin; MCHC = mean corpuscular hemoglobin concentration; MCV = mean corpuscular volume; PLT = platelet; RBC = red blood cell

The neonates diagnosed with ROP were further stratified into two cohorts: those necessitating treatment and those not requiring intervention. Among these groups, only RBC count (p=0.002) and HGB levels (p=0.001) exhibited statistically significant differences, while other hematological parameters and inflammatory markers demonstrated comparable profiles. Detailed data about CBC parameters, albumin levels, and inflammatory indices for both cohorts are provided in **Table 4**. A subsequent multivariate analysis revealed that neither RBC count nor HGB levels independently served as predictors for treatment necessity, as elucidated in **Table 5** through multivariate logistic regression analysis.

**Table 4:** Comparison of blood parameters between neonates requiring treatment for ROP and those not requiring treatment (Mean ± SD)

	Treatment (+) Neonates (n=41)	Treatment (-) Neonates (n=63)	p value*
RBC (10^3^ μL)	3.61 ± 0.69	4.07 ± 0.77	**0.002**
HGB (g/dL)	13.81 ± 2.49	15.57 ± 2.79	**0.001**
HCT (%)	50.33 ± 50.38	47.38 ± 8.48	0.649
MCV (fL)	118.17 ± 10.59	117.23 ± 10.30	0.652
MCHC (g/dL)	33.09 ± 2.06	33.25 ± 2.07	0.706
RDW (%)	18.33 ± 2.26	18.26 ± 2.07	0.861
WBC (10^3^ μL)	13.41 ± 13.67	13.53 ± 11.40	0.963
NEU (10^3^ μL)	7.40 ± 10.29	7.91 ± 8.84	0.790
LYM (10^3^ μL)	3.81 ± 3.00	4.24 ± 5.07	0.623
MONO (10^3^ μL)	1.19 ± 1.03	1.10 ± 0.86	0.604
PLT (10^3^ μL)	174.75 ± 98.73	186.61 ± 88.42	0.524
PCT (%)	0.14 ± 0.07	0.17 ± 0.21	0.390
ALBUMIN (g/dL)	2.84 ± 0.44	2.92 ± 0.51	0.409
NLR	1.91 ± 1.77	2.42 ± 2.40	0.245
PLR	56.19 ± 35.16	65.46 ± 53.14	0.286
SII	360.37 ± 396.48	498.25 ± 591.42	0.157
SIRI	3.10 ± 5.85	3.50 ± 5.35	0.718
PIV	643.95 ± 1493.77	764.45 ± 1339.82	0.669
HALP score	13.52 ± 20.96	14.98 ± 24.42	0.752

HALP score = hemoglobin, albumin, lymphocyte and platelet score; HGB = hemoglobin; HCT = hematocrit; LYM = lymphocyte; MCHC = mean corpuscular hemoglobin concentration; MCV = mean corpuscular volume; MONO = monocyte; NEU = neutrophil; NLR = neutrophil-to-lymphocyte ratio; PIV = pan-immune-inflammation value; platelet indices variation; PLR = platelet-to-lymphocyte ratio; PLT = platelet; PCT = plateletcrit; RBC = red blood cell; RDW = red cell distribution width; SII = systemic immune-inflammation index; SIRI = systemic inflammation response index; WBC = white blood cell

*Independent samples t-test; significant p-values are given in bold.

**Table 5:** Multivariate analysis of the predictors for treatment need for neonates diagnosed with ROP

Variable	OR	95% CI		p value
		Lower	Upper	
GA, weeks	0.912	0.682	1.219	0.534
BW, g	0.998	0.996	1.000	0.100
APGAR5	0.876	0.676	1.135	0.316
RBC (10^3^ μL)	1.713	0.417	7.045	0.456
HGB (g/dL)	0.719	0.488	1.061	0.097

BW = birth weight; GA = gestational age; HGB = hemoglobin; RBC = red blood cell

**Fig. 2:**
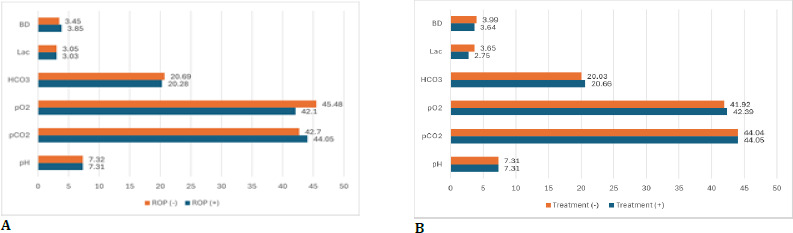
Comparison of ABG analysis results: (**A**) between neonates diagnosed with ROP and those not diagnosed, and (**B**) between neonates who required treatment and those who did not

No statistically significant differences were observed in any ABG parameters between neonates diagnosed with ROP and those without. Likewise, no significant disparities were noted in ABG analysis between infants necessitating treatment for ROP and those who did not. **Fig. 2** illustrates the comparison of these cohorts in terms of mean ABG parameter values.

## Discussion

In the current study, we utilized the initial 24-hour blood tests to examine the SII, SIRI, PIV, and HALP indexes, as well as NLR and PLR levels, as potential biomarkers of inflammation in patients with ROP. Additionally, we assessed individual CBC and ABG parameters, which reflect cellular and biochemical aspects of inflammation and oxygenation—both critical in ROP pathogenesis. Our findings revealed no significant differences in the SII, SIRI, PIV, HALP indexes, or NLR and PLR levels in the first 24-hour blood tests when comparing neonates with and without any stage of ROP. However, significant differences were observed in RBC, HGB, MCV, and MCHC values between neonates diagnosed with ROP and those without disease.

In our study, inflammatory cell counts, including neutrophils, lymphocytes, and monocytes, showed no significant differences between the ROP and no-ROP groups, nor between those requiring treatment and those who did not. Previous studies have reported significant variations in these cell counts both in early [16,26] (first day or week) and late [27] (first month) blood tests in ROP patients, while some of these studies [15,24,25] evaluated early and late blood results together and compared them. For example, Kurtul et al. [16] found significantly lower lymphocyte counts in the ROP group compared to the non-ROP group. At the same time, Ozturk et al. [15] reported no differences in neutrophil, lymphocyte, and platelet counts during the early period. Evaluating the first month CBC results, Akyuz Unsal et al. [27] indicated higher leukocytes and lower platelets in the ROP group compared to the non-ROP group, whereas Oruz et al. [25] observed lower neutrophils, higher lymphocytes, and higher platelets. A recent study reported no difference in monocyte counts between ROP infants requiring treatment and those who did not in the first week, though by the fourth week, differences emerged [28]. These discrepancies could be attributed to varying gestational ages among the study groups, as inflammatory cell counts change at different stages of neonatal development [29]. Typically, there is a postnatal decrease in neutrophils and monocytes, with an increase in lymphocytes. This increase in lymphocytes may stem from the fact that their maturation is not complete until the 32nd gestational week [30]. Another study observed that the prevalence of neutrophil abnormalities increased with immaturity, and as neonates matured, the range of values considered normal for neutrophils narrowed [31]. Therefore, differing gestational ages of study populations could significantly affect the results. Additionally, early neonatal blood counts, when performed with automated devices, may have low reliability [32,33]. Normoblasts, which are precursor red blood cells, can be mistakenly counted as leukocytes, thus compromising the reliability of early blood test results in neonates [34]. Moreover, factors such as late clamping of the umbilical cord, the use of antenatal steroids, and birth stress may influence the first 24-hour CBC results and contribute further to discrepancies [35].

Platelets are critical in angiogenesis, acting as carriers for key growth factors such as vascular endothelial growth factor, platelet-derived growth factor, and insulin-like growth factor-1 [14,36]. Thrombocytopenia may hinder normal retinal vascular development, potentially leading to uncontrolled retinal neovascularization due to a deficiency in these growth factors. Jensen et al. [36] established a correlation between low platelet counts and the incidence of type 1 ROP in zone 1 cases. At the same time, Keskek et al. [14] observed that decreased platelet levels during the first week of the neonatal period were associated with ROP. Our findings revealed significant differences in platelet counts between neonates with and without ROP; however, this distinction was not preserved in multivariate analysis. It is known that platelet levels initially decrease post-birth and begin to increase by the end of the first week. This temporal fluctuation could account for the diminished significance of platelet counts in multivariate analysis when adjusting for factors such as GA [37].

In our study, statistical differences were observed in RBC, HGB, MCV, and MCHC values between neonates with and without ROP in multivariate analysis. Although RBC and HGB levels differed significantly between neonates requiring treatment and those who did not, this significance disappeared in multivariate analysis. Pheng et al. [13] evaluated mean HGB levels in the first 6 weeks of life, finding significant differences between 31 babies with ROP and 31 without, only in the first week, even after adjusting for confounders. No significant differences were noted at birth or from weeks two to six. Also, initial significant differences in HGB levels between treatment-required ROP cases and controls were found at multiple points, but these were not substantial post-adjustment. Other studies have also indicated that anemia during the first few days of postnatal life is a significant risk factor for severe ROP [38,39]. Similar to our research, Lundgren et al. [40] found no significant difference in mean HGB levels during the first week of life between infants with ROP requiring treatment and controls after adjusting for confounders. Postnatally, HGB levels drop as the rate of RBC death exceeds their replacement rate. A physiological anemia occurs in all newborns, which is more pronounced in very preterm infants. Lower HGB levels may have a direct hypoxic effect on the retina, potentially contributing to ROP, which is closely related to retinal hypoxia [13]. The link between reduced HGB levels and ROP may also involve pathways related to nitric oxide (NO) [41]. HGB in RBCs not only transports and delivers oxygen but also captures NO, forming an Hb-NO complex [42]. NO primarily facilitates the relaxation of small resistance vessels, and excessive production at sites of inflammation can increase vasodilation and capillary permeability. NO also acts as a pro-inflammatory mediator, inducing inflammation due to overproduction in abnormal situations. Therefore, maintaining adequate HGB levels in RBCs is crucial to counterbalance NO at inflammation sites and help prevent ROP.

We observed that neonates with ROP exhibited higher MCV and MCHC values compared to those without ROP. In contrast, Akyuz Unsal et al. [27] reported that lower MCH was the most significant predictive marker in the 4th week of CBC analysis. We hypothesize that the discrepancies in MCHC values, calculated as hemoglobin/hematocrit, may be attributed to differing hematocrit levels between the two studies. Similarly, Fevereiro-Martins et al. [43] found that higher MCV in the first week of life correlates with ROP development in premature infants. This study also noted that erythroblasts, the precursors to mature red blood cells, were more prevalent in patients with ROP. The increased presence of erythroblasts might account for the elevated MCV observed. However, our study was unable to determine erythroblast counts.

We found no significant differences in ABG parameters between neonates with ROP and those without, or between those requiring treatment and those not requiring treatment for ROP. A study by Huang et al. [44], which analyzed early ABG results in preterm infants, noted that while hyperoxia and hypocarbia were uncommon, hypercarbia was frequent. However, these conditions did not correlate with adverse inpatient outcomes, including ROP or neonatal death. The ELGAN study suggested that infants exposed to high PCO2, low pH, and high PaO2 during the first few days of life may be at increased risk for developing severe ROP [45]. This indicates that the impact of ABG parameters should be assessed over an extended period rather than from initial results alone. Considering that blood gas levels can rapidly change with interventions such as oxygenation and fluid replacement, evaluating these parameters over a more extended period may offer a more reliable measure of outcomes than a single measurement.

So far, two studies have investigated the role of SII in ROP. One study, with a sample size of 92 ROP-positive and 103 ROP-negative neonates, found that the postnatal first-day NLR and SII values, and the postnatal first-month NLR, SII, and LMR values were significantly higher in terms of ROP development [25]. PLR values did not show significant differences between infants with and without ROP on either the postnatal first day or first month. However, in multivariate analyses conducted for the need for laser treatment, postnatal first-month PLR and SII values were identified as independent risk factors (OR: 0.951 and 1.011, respectively). The other study reported significantly different NLR, PLR, and SII at the first month between ROP-positive and ROP-negative neonates. Still, these values were not different during the first 24 hours [24]. Similarly, we found no significant differences in NLR, PLR, and SII during the first 24 hours.

To the best of our knowledge, this is the first study to explore the role of SIRI, PIV, and HALP score, derived from first-day blood test results, in predicting ROP development and treatment need. Compared to individual white blood cells and platelets, these indices are less influenced by the body’s physiological and pathological states, providing a more stable reflection of the overall inflammatory condition. In a study by Erdal et al. [46], SIRI and PIV values were higher in patients with pseudoexfoliation syndrome than in healthy controls. Another study involving 500 diabetic patients found that those with retinopathy exhibited significantly higher SII and SIRI values compared to those without retinopathy [22]. Additionally, a very recent study identified a lower HALP score as a significant risk factor for diabetic retinopathy, independent of other confounders [47]. Since all these studies were conducted in adult populations, the differences in the distribution and number of blood cells between pediatric and adult populations may explain the varying results when compared to our study.

Our study had several limitations. First, the study groups were not age- and gender-matched. This issue often arises in ROP studies, as ROP cases, particularly those requiring treatment, typically involve younger infants. To mitigate the effects of this demographic discrepancy, we employed multivariate analysis. Additionally, our study was retrospective and conducted at a single center. Despite these limitations, we successfully analyzed a substantial dataset comprising approximately 10 years of records, which is a notable strength of our research. Moreover, our study is the first to assess the utility of SIRI, PIV, and HALP scores, along with other parameters, in predicting the occurrence of ROP and the necessity for treatment. While including blood results from subsequent weeks could potentially enhance the study, as ROP generally manifests during this late period, such an extension was beyond our study’s scope. Our focus was on evaluating the predictive value of blood measurements within the first 24 hours.

## Conclusion

In conclusion, despite the established association between ROP and inflammation, our findings suggest that novel inflammatory parameters such as SII, SIRI, PIV, and HALP, derived from blood tests conducted within the first 24 hours, do not predict ROP diagnosis or treatment necessity. Therefore, it is crucial to consider potential confounders and sources of error when interpreting early blood results in neonates. Future prospective studies are warranted to conduct a comparative analysis of CBC and albumin levels, with collection at different stages of the neonatal period, to further investigate the role of these inflammatory markers in ROP.
